# Metabarcoding of soil environmental DNA to estimate plant diversity globally

**DOI:** 10.3389/fpls.2023.1106617

**Published:** 2023-04-18

**Authors:** Martti Vasar, John Davison, Mari Moora, Siim-Kaarel Sepp, Sten Anslan, Saleh Al-Quraishy, Mohammad Bahram, C. Guillermo Bueno, Juan José Cantero, Ezequiel Chimbioputo Fabiano, Guillaume Decocq, Rein Drenkhan, Lauchlan Fraser, Jane Oja, Roberto Garibay-Orijel, Inga Hiiesalu, Kadri Koorem, Ladislav Mucina, Maarja Öpik, Sergei Põlme, Meelis Pärtel, Cherdchai Phosri, Marina Semchenko, Tanel Vahter, Jiři Doležal, Aida M. Vasco Palacios, Leho Tedersoo, Martin Zobel

**Affiliations:** ^1^Institute of Ecology and Earth Sciences, University of Tartu, Tartu, Estonia; ^2^Zoology Department, College of Science, King Saud University, Riyadh, Saudi Arabia; ^3^Department of Ecology, Swedish University of Agricultural Sciences, Uppsala, Sweden; ^4^Instituto Multidisciplinario de Biología Vegetal, Universidad Nacional de Córdoba, CONICET, Córdoba, Argentina; ^5^Departamento de Biología Agrícola, Facultad de Agronomía y Veterinaria, Universidad Nacional de Río Cuarto, Córdoba, Argentina; ^6^Department of Wildlife Management and Ecotourism, University of Namibia, Katima Mulilo, Namibia; ^7^Ecologie et Dynamique des Systèmes Anthropisés (EDYSAN, UMR CNRS 7058), Jules Verne, University of Picardie, Amiens, France; ^8^Institute of Forestry and Engineering, Estonian University of Life Sciences, Tartu, Estonia; ^9^Department of Natural Resource Sciences, Thompson Rivers University, Kamloops, BC, Canada; ^10^Instituto de Biología, Universidad Nacional Autónoma de México, Ciudad de México, Mexico; ^11^Iluka Chair in Vegetation Science and Biogeography, Harry Butler Institute, Murdoch University, Perth, WA, Australia; ^12^Department of Geography & Environmental Studies, Stellenbosch University, Stellenbosch, South Africa; ^13^Center of Mycology and Microbiology, University of Tartu, Tartu, Estonia; ^14^Department of Biology, Nakhon Phanom University, Nakhon Phanom, Thailand; ^15^Institute of Botany, The Czech Academy of Sciences, Třeboň, Czechia; ^16^Faculty of Science, University of South Bohemia, České Budějovice, Czechia; ^17^Grupo de Microbiología Ambiental y Grupo BioMicro, Escuela de Microbiología, Universidad de Antioquia UdeA, Medellín, Colombia; ^18^Department of Botany, University of Tartu, Tartu, Estonia

**Keywords:** distribution, diversity, environmental DNA, molecular methods, plant, soil, TRNL

## Abstract

**Introduction:**

Traditional approaches to collecting large-scale biodiversity data pose huge logistical and technical challenges. We aimed to assess how a comparatively simple method based on sequencing environmental DNA (eDNA) characterises global variation in plant diversity and community composition compared with data derived from traditional plant inventory methods.

**Methods:**

We sequenced a short fragment (P6 loop) of the chloroplast trnL intron from from 325 globally distributed soil samples and compared estimates of diversity and composition with those derived from traditional sources based on empirical (GBIF) or extrapolated plant distribution and diversity data.

**Results:**

Large-scale plant diversity and community composition patterns revealed by sequencing eDNA were broadly in accordance with those derived from traditional sources. The success of the eDNA taxonomy assignment, and the overlap of taxon lists between eDNA and GBIF, was greatest at moderate to high latitudes of the northern hemisphere. On average, around half (mean: 51.5% SD 17.6) of local GBIF records were represented in eDNA databases at the species level, depending on the geographic region.

**Discussion:**

eDNA trnL gene sequencing data accurately represent global patterns in plant diversity and composition and thus can provide a basis for large-scale vegetation studies. Important experimental considerations for plant eDNA studies include using a sampling volume and design to maximise the number of taxa detected and optimising the sequencing depth. However, increasing the coverage of reference sequence databases would yield the most significant improvements in the accuracy of taxonomic assignments made using the P6 loop of the trnL region.

## 1 Introduction

High-throughput sequencing of environmental DNA (eDNA) from multiple species in parallel (metabarcoding) allows researchers to gather vast amounts of information about the organisms present in ecosystems and is already an important tool used to describe, model, and predict biodiversity in space and time (Cristescu and Hebert, 2018; Leff et al., 2018; Calderón-Sanou et al., 2020). Plant diversity detected from eDNA in the soil has been found to closely mirror plant taxonomic and growth form diversity estimated from conventional above-ground surveys (Yoccoz et al., 2012; Edwards et al., 2018). Thus, it appears possible to gain a detailed understanding of plant diversity at an unprecedented scale using a standardised metabarcoding approach, while overcoming the problems of uneven regional distribution and the limits of taxonomic expertise that limit traditional vegetation survey data, especially when working with little-studied floras. It is also important to note that eDNA reflects community composition both at present and in the recent past; up to 40% of the DNA extracted from soil samples may originate from extracellular material, i.e., remnants of dead organisms (Carini et al., 2016). While this means that eDNA does not provide the same strict temporal snapshot provided by traditional methods, the more stable view it provides is arguably at least as valuable as a source of information for understanding the composition of biotic communities.

Yet there have been relatively few attempts to describe large-scale variation in plant diversity using eDNA metabarcoding. Willerslev et al. (2014) and Wang et al. (2021) used permafrost samples to address ancient circumpolar plant diversity, while Barnes et al. (2022) used modern soil samples to describe variation in Danish plant communities along major environmental gradients. In all cases, however, good reference sequence databases were available for species in the regional flora. We aimed to assess the value of eDNA in describing global-scale variation in plant community composition. Floristic expertise is patchy globally, and there are large areas where the floras are virtually unknown (Brummitt et al., 2021), even in otherwise well studied regions (Mokany et al., 2022). Existing information about global patterns of plant diversity comes in the form of traditional survey observations (such as those collated by the Global Biodiversity Information Facility, GBIF) and global extrapolations of diversity (Kreft and Jetz, 2007; Cai et al., 2022). Metabarcoding might thus provide a uniquely complete and systematic empirical insight into large-scale variation in plant diversity.

We collected plant eDNA in soil from an existing global sampling network (Vasar et al., 2022) and sequenced the P6 loop of the plastid trnL (UAA) intron (Taberlet et al., 2007; Mallott et al., 2018; Barnes et al., 2022) to assess variation in plant diversity and determine its most critical climatic drivers. To place the eDNA results in the context of macroecology, we compare them with existing information about large-scale variation in the occurrence and abundance of plant species derived from available empirical (GBIF) and extrapolated plant diversity data (Kreft and Jetz, 2007; Cai et al., 2022). We also estimate the coverage of recorded plant species and families in trnL sequence databases and consider how these influence estimated diversity patterns.

## 2 Materials and methods

### 2.1 Global soil samples

We used soil samples collected from 325 locations worldwide. The sampling locations and sample collection protocol are described by Vasar et al. (2022). The sampling protocol was designed, following Taberlet et al. (2012), to minimise the influence of local heterogeneity by incorporating soil from multiple cores. The DNA mixture extracted from replicate soil samples is thus expected to represent collective local biodiversity as closely as possible. Briefly, at each sampling location, a site that was little disturbed by human activities was identified. About 20 g of topsoil (1–5 cm) was collected from up to the 40 points within an approximately 50 × 50 m sampling area. For further analysis, the samples were pooled per site, dried, and homogenised. For molecular analysis, a 2 g subsample of soil was collected from the pooled sample.

### 2.2 Molecular methods

DNA was extracted from 2 g of dried soil using the PowerMax Soil DNA Isolation Kit. The short fragment of the trnL (UAA) intron (the P6 loop, 10-143 bp) was amplified using primers trnL-g (5′-GGGCAATCCTGAGCCAA-3′) and trnL-h (5′-CCATTGAGTCTCTGCACCTATC-3′) (Taberlet et al., 2007) for identification of plants. The P6 loop of the trnL intron is short enough to allow amplification of degraded plant DNA from soil samples (Taberlet et al., 2007). Thermal cycling included an initial hot-start denaturation at 95°C for 15 min, 35 cycles of denaturation for 30 s at 95°C, annealing for 30 s at 55°C, elongation for 10 s at 72°C and storage at 4°C. Barcode selection and PCR product preparation follow Vasar et al., 2022. Libraries were sequenced on the Illumina MiSeq platform, using a 2 × 150 bp paired‐end sequencing approach at Asper Biogene (Tartu, Estonia). Raw reads from this Targeted Locus Study have been deposited in the NCBI SRA (BioProject PRJNA659159).

### 2.3 Environmental variables

Estimates of mean annual temperature (MAT), mean annual precipitation (MAP) and precipitation seasonality (SeaPrec) at sample locations were taken from the CHELSA database (Karger et al., 2017). These climatic variables are expected to be important drivers of plant communities (Sabatini et al., 2022) and have large-scale dynamics making them relevant to plots of variable size (GBIF, Kreft and Jetz, 2007 and Cai et al., 2023). Only variables that have annual measurements were selected from the CHELSA database to remove the possible effect of seasonality.

### 2.4 Bioinformatics

Plant trnL data were analysed using the gDAT pipeline (Vasar et al., 2021). Combining paired-end reads was conducted with FLASh (v1.2.1071, Magoč and Salzberg, 2011) using the default parameters (at least 10bp overlap with 75% identity) and enabling the option for outie alignments, which allows overlaps at the beginning of reads, rather than exclusively at the end. As the insert sizes (when outie alignment occurs) are shorter than MiSeq read length, sequences result in palindrome reads where adapter read-through occurs (Bolger et al., 2014). Adapters were removed from both ends using pipeline built-in functionality. Reads were demultiplexed, allowing 1 mismatch for the barcode and primer for both pairs. Only pairs where both reads had an average quality score of >30 were retained (after the removal of barcode sequences). Reads < 20bp (excluding primer length) were removed as they contain very little information for defining OTUs, assigning taxonomy and detecting potential chimeric sequences. Orphan reads (paired end reads that did not meet the conditions for combination and cleaning) were removed from the analyses, leaving 2,050,981 cleaned sequences. The VSEARCH (v2.14.166, Rognes et al., 2016) chimera filtering algorithm was used to remove putative chimeric reads in *de novo* mode, yielding 2,042,297 chimera-free sequences. Reads were clustered with VSEARCH at 97% identity into 32,127 OTUs (excluding singletons). Representative sequences (OTU centroids) for each non-singleton OTU were taxonomically classified using a BLASTn (v2.11.0+, Camacho et al., 2009) search against the INSDC (International Nucleotide Sequence Database Collaboration, status October 2022) database using up to 300 best hits for each query sequence, which were used to identify the lowest common ancestor by the pipeline based on the following criteria: > 80% alignment length; identification for species level > 97% identity, for genera > 95% and for family > 90%; consensus achieved when 51% of hits shared the same taxonomy at each rank. A total of 29,271 OTUs were identified as plants; 43 OTUs were identified as Bacteria, Fungi or Metazoa, which, along with OTUs that could not be identified, were removed from the data set prior to analysis.

### 2.5 Comparison datasets

The Global Biodiversity Information Facility (GBIF) collates a variety of information about organisms and their geographical locations. We took information about the distributions of vascular plant species in GBIF from Tamme et al. (2021) including 156,200,298 georeferenced vascular plant occurrences for 289,295 species (available for download: https://doi.org/10.15468/dl.4nqoev). The dataset was cleaned by the authors to group the species with synonym entries, reducing occurrences to 266,074 species, which were normalised into hexagonal cells using dggridR package in R (Bousquin, 2021), producing 65,612 equally sized grids cells (cell area of approximately 7,774 km2) worldwide.

We also took empirical estimates of plant alpha diversity worldwide from the information provided by Kreft and Jetz (2007), which presented geographic patterns of global vascular plant diversity at the species level. It used an exhaustive dataset of 1032 regions worldwide to generate expert-opinion-based continental to global maps of plant species richness. The dataset provides a cokriging estimate (cokrig), which was used as a richness estimate for our study. Finally, we considered the recently updated empirical estimates of plant alpha diversity worldwide produced by the Cai et al. (2022) (hereafter Cai) who used machine learning to improve the global models. We compared Cai with Kreft & Jetz dataset to assess the improvements.

### 2.6 Statistics

To normalise eDNA sequence count data, we implemented the variance stabilising transformation (in R package DESeq2 v1.28.175), as suggested by McMurdie and Holmes (2014). The method uses fitted dispersion-mean relationships to normalise data with respect to sample size (sequencing depth of individual samples) and variance.

For each eDNA sampling location, the intersecting cell from the GBIF, Kreft & Jetz dand Cai et al., 2022 datasets was identified. Multiple data subsets were then generated with the following criteria: eDNA reads - raw read counts from eDNA sequences; eDNA c97% - raw reads clustered at 97% similarity threshold and singletons removed; eDNA VST - clustered reads at 97% similarity, variance stabilised transformed; eDNA family - INSDC BLASTn hits extracted at family level; eDNA species - INSDC BLASTn hits extracted at species level; Kreft & Jetz – Kreft & Jetz cokrig value; GBIF family - GBIF dataset grouped at family level; GBIF species - GBIF dataset grouped at species level; Cai – Cai et al., 2022 machine learning predicted richness using ensemble prediction. The data subsets were then correlated using Pearson’s correlation and plotted (using chart.Correlation from R package PerformanceAnalytics). The significance of coefficient estimates was estimated using Dutilleul et al.’s (1993) correction for spatial autocorrelation. The correction was implemented using a version of the modified.ttest function from the R package SpatialPack (Vallejos et al., 2020) that was adapted to incorporate great circle distances, i.e., distances over the surface of a sphere such as the Earth; the modified function is available from Davison et al. (2022).

Climatic drivers of eDNA and GBIF communities were identified using distance-based redundancy analysis (dbRDA; vegan package in R, Oksanen et al., 2013). Variation in sample distance matrices (Bray-Curtis distance following variance stabilising transformation) was modelled against all measured climatic variables. The significance of the effects was measured using permutation (n=999). Sampling locations were assigned to biogeographic realms following Olson et al. (2001).

We predicted the global distribution of the four different diversity measures (eDNA, GBIF, Kreft & Jetz and Cai) using generalised additive models (GAMs) and the spline-over-the-sphere algorithm in the R package mgcv, with the method ‘sos.smooth’ and the default arguments except k = 90 (Wood, 2003). This model can predict smooth variation in diversity values over the globe without producing edges. To make the spatial patterns comparable, we used for GBIF, Kreft & Jetz and Cai data only the subset of cells that intersected the eDNA sampling locations.

We estimated the availability of plant taxon reference sequences in the INSDC by searching “trnL[Gene Name] OR tRNA[Gene Name] OR Leu[Gene Name]”. We recorded 138,286 sequences, including 64,513 plant species and 529 plant families (accessed on October 2022). For each sampling location, we estimated the fraction of recorded GBIF families and species represented in INSDC; and the fractions of eDNA reads and OTUs getting a match against families and species in INSDC. We also calculated the overlap of family- and species-level taxa recorded in eDNA and GBIF. We then used GAM models, as described above, to assess geographical variation in the match between plant occurrence data (eDNA and GBIF) and reference sequence data.

## 3 Results

### 3.1 Comparison of eDNA richness with GBIF, Kreft & Jetz and Cai richness estimates

**Figure 1:**
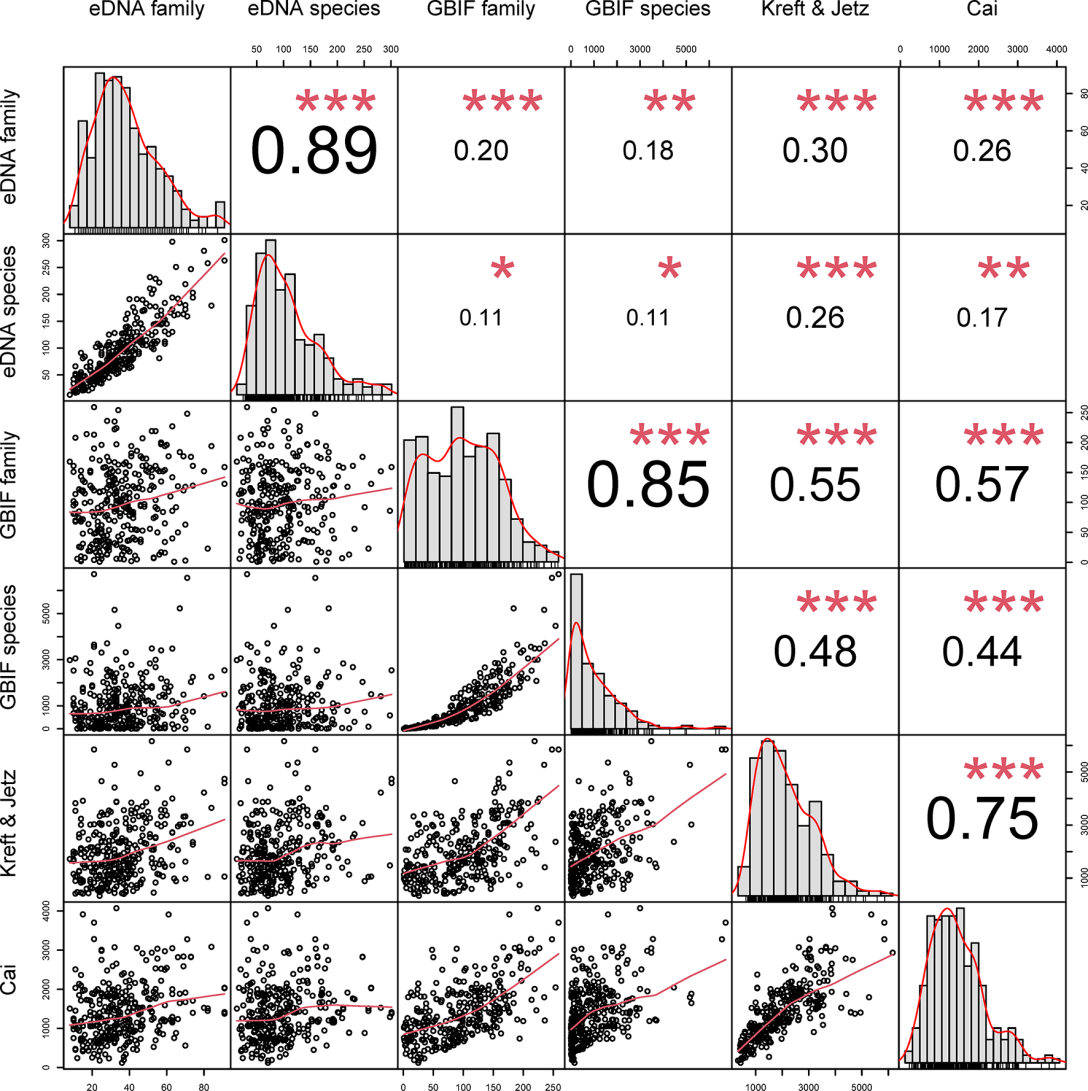
Correlations between eDNA, GBIF, Kreft & Jetz and Cai datasets. Numbers show correlation strength and direction, red asterisks show significance (*p < 0.05; **p < 0.01; ***p < 0.001).

Richness estimated using eDNA was weakly-to-moderately positively correlated with the corresponding estimates derived from GBIF (family: r = 0.20, p < 0.001; species: r = 0.11, p = 0.045), Kreft & Jetz (eDNA family: r = 0.30, p < 0.001; eDNA species: r = 0.25, p < 0.001) and Cai (eDNA family: r = 0.26, p < 0.001, eDNA species: r = 0.17, p < 0.01) at both the species and family level, with family-level correlations systematically stronger (**Figure 1**, **Figure S1**). The eDNA metrics were highly correlated with each other (r = 0.50-0.89, all p < 0.001), as were the GBIF metrics (r = 0.86, p < 0.001), and Cai and Kreft & Jetz (r = 0.74, p < 0.001). eDNA had a stronger correlation with Kreft & Jetz than with GBIF and Cai. Different taxonomic levels of GBIF were positively correlated with Kreft & Jetz (GBIF family: r = 0.55, p < 0.001; GBIF species level: r = 0.48, p < 0.001). Compared with Kreft & Jetz, Cai was more strongly correlated with GBIF family (r = 0.57, p < 0.001) but more weakly correlated with GBIF species (r = 0.44, p < 0.001). Pearson’s correlations with significance values corrected for spatial autocorrelation are shown in **Table S1**.

### 3.2 GAM and dbRDA analyses

**Figure 2:**
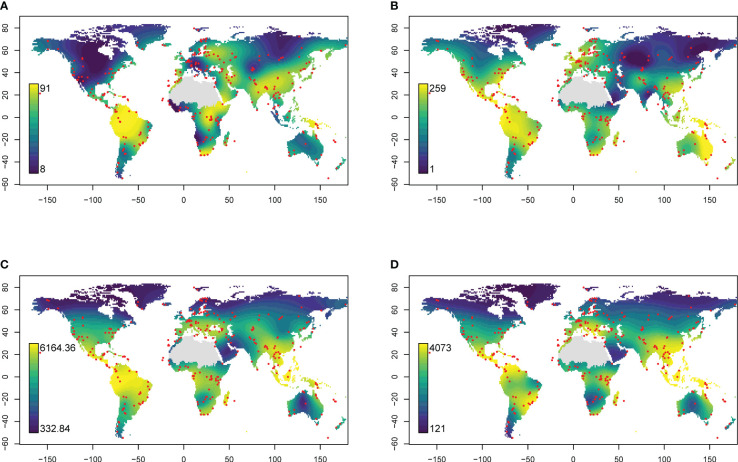
Global distribution of **(A)** eDNA family richness, **(B)** GBIF family richness, **(C)** Kreft & Jetz estimate of richness and **(D)** Cai estimates of richness predicted using generalised additive models. Red points indicate sampling locations. The Sahara region was excluded from interpolations because of insufficient sampling.

**Figure 3:**
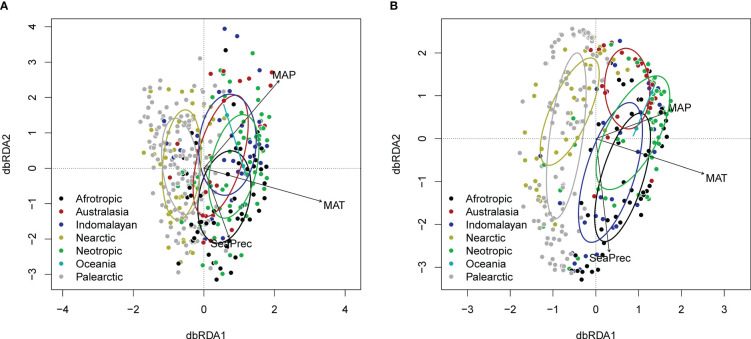
dbRDA plot (distance-based redundancy analysis) showing effects climatic variables (CHELSA) and biogeographic realm on **(A)** eDNA and **(B)** GBIF family level composition (following variance stabilising transformation). Ellipses indicate 1 standard deviation around the centroids for different biogeographic realms.

Generalised additive models identified similar global hotspots and coldspots of richness for all four datasets (**Figure 2**). dbRDA analysis revealed similar relationships between eDNA- or GBIF-derived plant family composition patterns and climatic factors, with colder and warmer regions generally clustering separately (**Figure 3**).

### 3.3 eDNA taxon representation in GBIF and INSDC

The annotated eDNA reads comprised 412 families and 4550 species, while 496 families and 106,490 species were recorded in the intersecting cells of the GBIF dataset. Altogether 304 families were shared between sets, while 108 and 192 were unique for eDNA and GBIF, respectively. In turn, 2663 species were shared between sets, while 1887 and 103,827 species were uniquely recorded in eDNA and GBIF, respectively.

**Figure 4:**
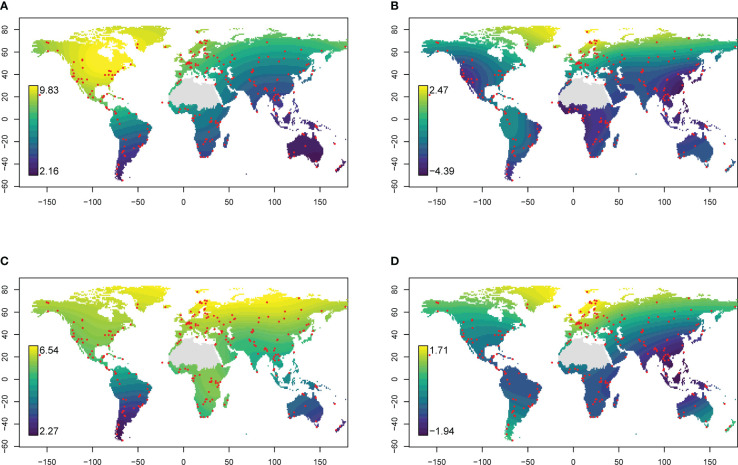
Relative success of taxonomic assignment of eDNA reads (trnL P6 loop). Values are the logarithm of the ratio between the number of reads **(A, B)** or OTUs **(C, D)** getting a match against trnL intron sequence data in INSDC at family **(A, C)** or species **(B, D)** level and the number not getting a match. Higher values indicate greater proportions of reads or OTUs getting a match. Red points indicate sampling locations. Global predictions are the result of a generalised additive model. The Sahara region was excluded from interpolations because of insufficient sampling.

The success of taxonomic assignment varied between different geographic regions. The highest rate of species-level assignment was 82.3%, with 82 out of 99 OTUs identified in a sample; the lowest rate was 5.6%, with only 2 OTUs out of 36 OTUs identified in a sample. In general the proportion of OTUs being assigned to species level was 38.7%. The proportion of reads and OTUs being successfully matched against species represented in INSDC was notably high in the peri-arctic region and lower elsewhere (**Figure 4**); the family-level pattern was less pronounced but still characterised by successful assignment at northern latitudes (**Figure 4**). Among GBIF occurrence records, there was similar geographic variation in the proportion of taxa (families and species) represented among trnL sequence data in INSDC (**Figure S2**). At the species level, representation in INSDC was highest at northern latitudes and decreased towards the equator, remaining low in the Southern Hemisphere. Among GBIF families, representation was highest in Europe, North America, and Australia. The proportion of eDNA reads and OTUs representing species and families that were also present in the corresponding GBIF data also exhibited substantial geographic variation: family-level matching was highest in Europe and North America with the strongest discrepancy in parts of Asia; species-level matching was highest at high northern latitudes (**Figure S3**).

## 4 Discussion

We used soil eDNA metabarcoding to characterise variation in plant community richness and composition across the globe. We show that the short fragment of the trnL region produces richness measures that significantly correlate with independent estimates based on empirical data collation and extrapolation. Thus, we can confirm that, despite the small number of sample points, eDNA trnL gene sequencing data accurately represent global patterns in plant diversity and composition and thus can provide meaningful input for large-scale studies of vegetation and plant communities.

However, there are important issues and trade-offs to consider when using eDNA sequencing. First, using eDNA requires careful planning, with marker region and primer selection tailored to the characteristics of the target organism. Although taxonomic resolution increases with marker length and variability, the DNA in environmental samples is often, to a certain degree, degraded and present at low concentrations. This means that shorter DNA markers, such as the P6 loop of the plastid trnL (UAA) intron (Taberlet et al., 2007; Mallott et al., 2018; Barnes et al., 2022), may be most effective for use in plant biodiversity assessments across a range of environmental conditions. For example, the short fragments of the P6 loop were successfully used when amplifying DNA in sediment, where < 12k year old samples yielded reads of similar length compared with the present day samples (Rijal et al., 2021). Local-scale studies of eDNA using the trnL gene have recorded strong correlations between plant community taxon composition based on vegetation plots and by metabarcoding of soil DNA (Yoccoz et al., 2012; Edwards et al., 2018; Calderón-Sanou et al., 2020). However, in many cases, the molecular approach could not identify plants at the species level because of the low taxonomic resolution of the short marker region (Foster et al., 2021). Another notable challenge is the incompleteness of reference databases (Kolter and Gemeinholzer, 2021), meaning that even when sequencing data with species-level resolution are achievable, taxonomic assignment with the same resolution may not be possible (Barnes et al., 2022). At the same time, local (Hiiesalu et al., 2012; Yoccoz et al., 2012; Hiiesalu et al., 2014; Hiiesalu et al., 2021; Sepp et al., 2021) and regional (Barnes et al., 2022) studies have shown that trnL can be a viable option for approximately species-level identification where existing species are well known, and a custom curated reference database can be built. Intriguingly, we found that eDNA richness estimates that are independent from potential annotation biases (i.e. there were 2849 OTUs and 33,309 sequences without taxonomic annotation) exhibited weaker correlations with the estimates from global biodiversity databases compared with taxonomically-assigned eDNA richness. This might reflect biases in any of the data sources, such as artefactual diversity among the eDNA data that was taxonomically unassigned, or similar biases in the taxonomic and geographic coverage of INSDC and GBIF databases such that taxonomically-assigned eDNA data and GBIF diversity data detect the same incomplete fraction of true diversity.

Our analysis showed that the efficiency of taxonomic assignment of eDNA reads and OTUs varied systematically across the globe, with many more reads and OTUs from high northern latitudes getting a species- or (to a lesser degree) family-level match. A comparison of GBIF records with taxa represented by trnL data in INSDC showed a similar pattern. It thus appears that trnL data held in INSDC are likely to be most taxonomically complete in northern Europe and North America but relatively sparse elsewhere. This geographical variation in sequencing data completeness likely reflects the intensity of efforts to sequence local floras, particularly the effort by Yoccoz et al. (2012); Edwards et al. (2018), Wang et al. (2021) and others to sequence much of the peri-arctic flora. Northern latitudes also showed the greatest taxon overlap between eDNA and GBIF records. It is possible that this reflects differences in landscape-level beta diversity among the very different spatial scales at which the two approaches sample alpha diversity. For example, the small scale eDNA approach may generate taxon lists that match well with the large-scale GBIF data from regions where there is low beta diversity (e.g. boreal forests) but poorly from landscapes with high beta diversity (e.g. tropical biomes). On the other hand, it seems likely that GBIF exhibits similar geographical variation in coverage to that apparent in the INSDC sequencing set. This highlights regions where occurrence and sequence data are systematically lacking and the need to assess patterns emerging from database records critically. An analogous situation concerning the representation of tree species in databases has been described by Keppel et al. (2021).

Stronger correlations between eDNA and GBIF at the family compared with the species level reflect the relatively low discriminatory power of the molecular approach, owing to the short length of the amplicon. For instance, this marker does not distinguish all species in certain large taxonomic groups, including diverse and widely distributed families such as Asteraceae and Poaceae (Hiiesalu et al., 2014; Träger et al., 2019). When sequencing the trnl P6 loop, the amplicon length is shorter than typical read length, and improper handling of sequence data can result in reduced data capture and a loss of taxonomic resolution. Such short-read pairs are often palindrome reads (forward and reverse reads are exact complement reads) containing adapter sequences at the end of the sequence, which, if not explicitly identified, can be incorrectly handled by bioinformatics pipelines such that the paired-end reads are not successfully combined and thus discarded. This affects the overall read count that goes into the final analysis, which can influence estimates of richness and composition. In principle, ITS2 primers could be preferable to the trnL primers since they recover a higher proportion of known richness, but trnL primers are likely more effective than ITS2 primers when the template DNA is highly degraded (Barnes et al., 2022). The use of multiple DNA marker regions might increase resolution and the number of plant reference sets available for taxonomy assignment (see Leff et al., 2018; Banchi et al., 2020; Bell et al., 2017), but this increases the cost and complexity of the analysis and therefore may not be feasible.

Local and regional studies have shown that when the aim is not to identify species but rather to characterise plant community differences and dynamics, an approach based on sequencing the trnL P6 loop is viable and rewarding (Sepp et al., 2021; Barnes et al., 2022). Despite the variety of vegetation worldwide and vastly different levels of information concerning different regions, we show that the same applies to studying the global flora. There was good agreement between the large-scale patterns described by eDNA and patterns described using information from GBIF, Kreft and Jetz (2007) or Cai et al. (2022), despite the low sample size used to characterise the global patterns of plant diversity. Thus, in detecting macroecological patterns, an approach based on plant eDNA sequencing can potentially be as informative as traditional plant surveys. Further research may inform about approaches to optimise the coverage and taxonomic resolution of eDNA analysis – for example, concerning the volume and spatial extent of sampling; or sequencing and bioinformatics approaches that maximise data volume and capture of taxonomic signal. However, the low representation of plant species in reference databases is a major limitation in workflows using taxonomy assignment and appears likely to remain so for the foreseeable future. Increasing the taxonomic and geographic coverage of reference sequences in databases remains an urgent and important task.

## Data availability statement

The datasets presented in this study can be found in online repositories. The names of the repository/repositories and accession number(s) can be found below: https://www.ncbi.nlm.nih.gov/bioproject/PRJNA659159.

## Author contributions

Conceptualisation, MV, JD, MM, MZ and S-KS. methodology, MV, JoD. software, MV. formal analysis, MV. investigation, MM, MV, MZ, MP. resources, SA-Q, MZ, LT. data curation, SA, MB, CB, JC, JiD, EF, GD, RD, LF, RG-O, IH, KK, CP, MS, TV, LM, SP, MÖ, AP. writing—original draft preparation, MV, MZ, MM, JoD. writing—review and editing, JoD. visualisation, MV. project administration, MZ, LT, SA-Q, MM. All authors have read and agreed to the published version of the manuscript. All authors contributed to the article and approved the submitted version.
