# Supplementary material for: Metabarcoding of soil environmental DNA to estimate plant diversity globally
Source: Front Plant Sci. 2023 Apr 18;14:1106617. doi: 10.3389/fpls.2023.1106617 (PMC10151745; doi:10.3389/fpls.2023.1106617)
Supplement: Supplementary file 1 [file DataSheet_1.pdf]

Supplementary Material

Metabarcoding of soil environmental DNA to estimate plant diversity globally

Martti Vasar*, John Davison, Mari Moora, Siim-Kaarel Sepp, Sten Anslan, Saleh Al-Quraishy, Mohammad Bahram, C. Guillermo Bueno, Juan José Cantero, Ezequiel Chimbioputo Fabiano, Guillaume Decocq, Rein Drenkhan, Lauchlan Fraser, Jane Oja, Roberto Garibay-Orijel, Inga Hiiesalu, Kadri Koorem, Ladislav Mucina, Maarja Öpik, Sergei Põlme, Meelis Pärtel, Cherdchai Phosri, Marina Semchenko, Tanel Vahter, Jiři Doležal, Aida M. Vasco Palacios, Leho Tedersoo, Martin Zobel

*** Correspondence:**
Martti Vasar
martti.vasar@ut.ee

1 Supplementary Figures and Tables

1.1 Supplementary Tables

Supplementary Table S1. Pearson's correlation (corrected for spatial autocorrelation) between eDNA, GBIF and Kreft & Jetz datasets. The same comparison pairs are shown in Fig 1. Significant values are marked in bold.

Dataset 1	Dataset 2	F-statistics	DF	Correlation	P-value
eDNA reads	eDNA c97%	621.814	233.982	0.852	< **0.001**
eDNA reads	eDNA VST	261.543	304.948	0.68	< **0.001**
eDNA reads	eDNA family	164.423	235.577	0.641	< **0.001**
eDNA reads	eDNA species	265.106	220..156	0.739	< **0.001**
eDNA reads	Kreft & Jetz	3.431	196.787	0.131	0.066
eDNA reads	GBIF family	0.047	276.809	0.0131	0.828
eDNA reads	GBIF species	0.361	294.102	0.035	0.548
eDNA reads	Cai	0.791	299.24	0.51	0.375
eDNA c97%	eDNA VST	356.792	295.295	0.74	< **0.001**
eDNA c97%	eDNA family	275.0538	224.176	0.742	< **0.001**
eDNA c97%	eDNA species	674.91	205.15	0.876	< **0.001**
eDNA c97%	Kreft & Jetz	2.521	249.426	0.1	0.114
eDNA c97%	GBIF family	0.0749	363.968	0.014	0.785
eDNA c97%	GBIF species	0.92	301.44	0.055	0.338
eDNA c97%	Cai	0.12	307.737	0.02	0.729
eDNA VST	eDNA family	99.368	301.088	0.498	< **0.001**
eDNA VST	eDNA species	168.534	293.399	0.604	< **0.001**
eDNA VST	Kreft & Jetz	0.3	363.266	0.029	0.584
eDNA VST	GBIF family	0.001	403.571	-0.002	0.973
eDNA VST	GBIF species	0.127	347.514	-0.0191	0.722
eDNA VST	Cai	0.077	319.78	0.016	0.782
eDNA family	eDNA species	717.803	195.608	0.887	< **0.001**
eDNA family	Kreft & Jetz	11.917	119.516	0.301	< **0.001**

eDNA family	GBIF family	7.023	171.881	0.198	**0.009**
eDNA family	GBIF species	6.676	206.143	0.177	**0.011**
eDNA family	Cai	24.428	303.285	0.273	< **0.001**
eDNA species	Kreft & Jetz	8.921	129.044	0.254	**0.003**
eDNA species	GBIF family	2.38	200.894	0.108	0.125
eDNA species	GBIF species	2.725	222.414	0.11	0.1
eDNA species	Cai	9.606	310.456	0.173	**0.002**
Kreft & Jetz	GBIF family	12.523	29.136	0.548	< **0.001**
Kreft & Jetz	GBIF species	14.573	47.959	0.483	< **0.001**
Kreft & Jetz	Cai	218.237	180.703	0.74	< **0.001**
GBIF family	GBIF species	132.901	48.939	0.855	< **0.001**
GBIF family	Cai	117.608	240.785	0.573	< **0.001**
GBIF species	Cai	66.887	275.376	0.442	< **0.001**

1.2 Supplementary Figures

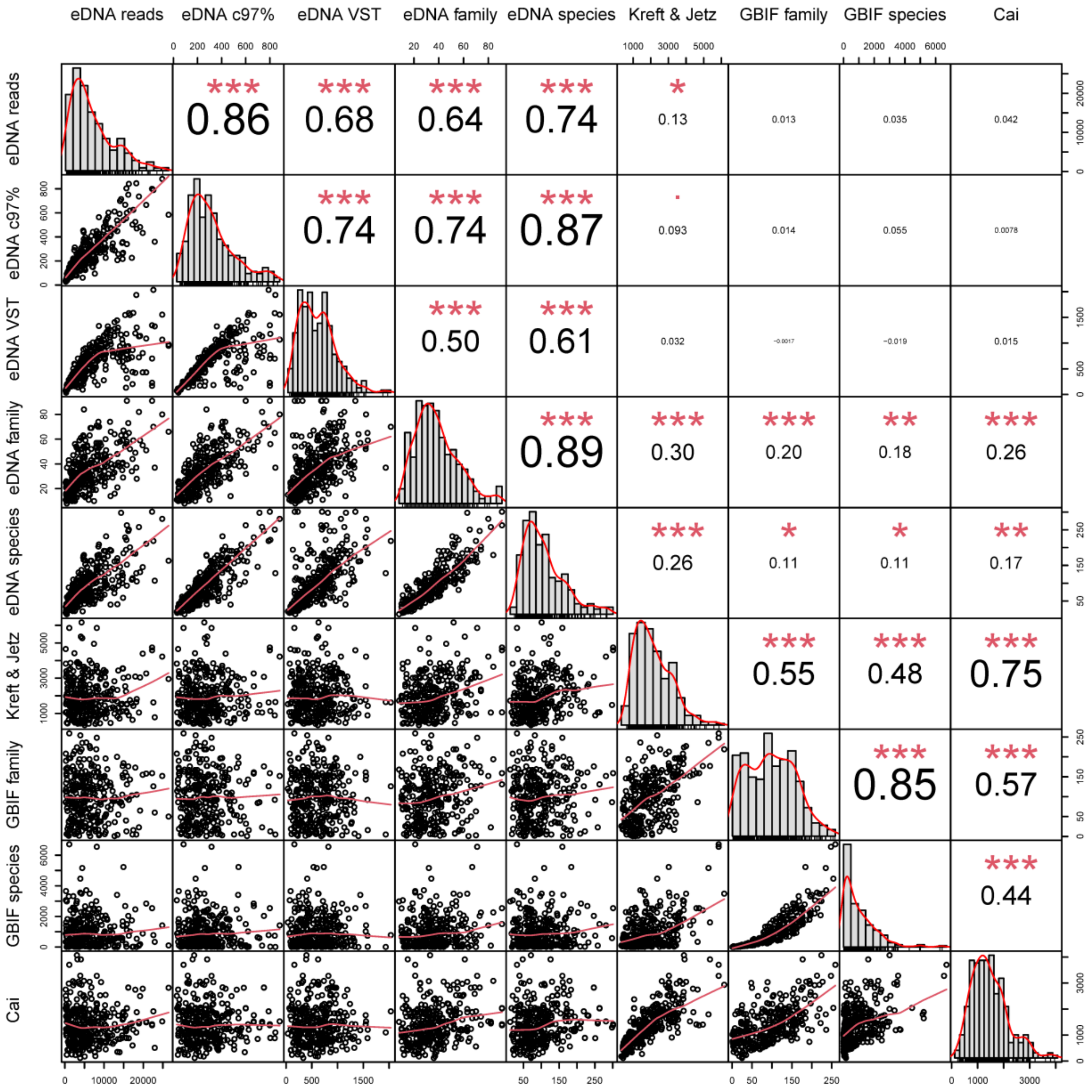

Supplementary Figure S1. Correlations between eDNA, GBIF, Kreft & Jetz and Cai datasets. Numbers show correlation strength and direction, red asterisks show significance (* – p < 0.05; ** – p < 0.01; *** – p < 0.001).

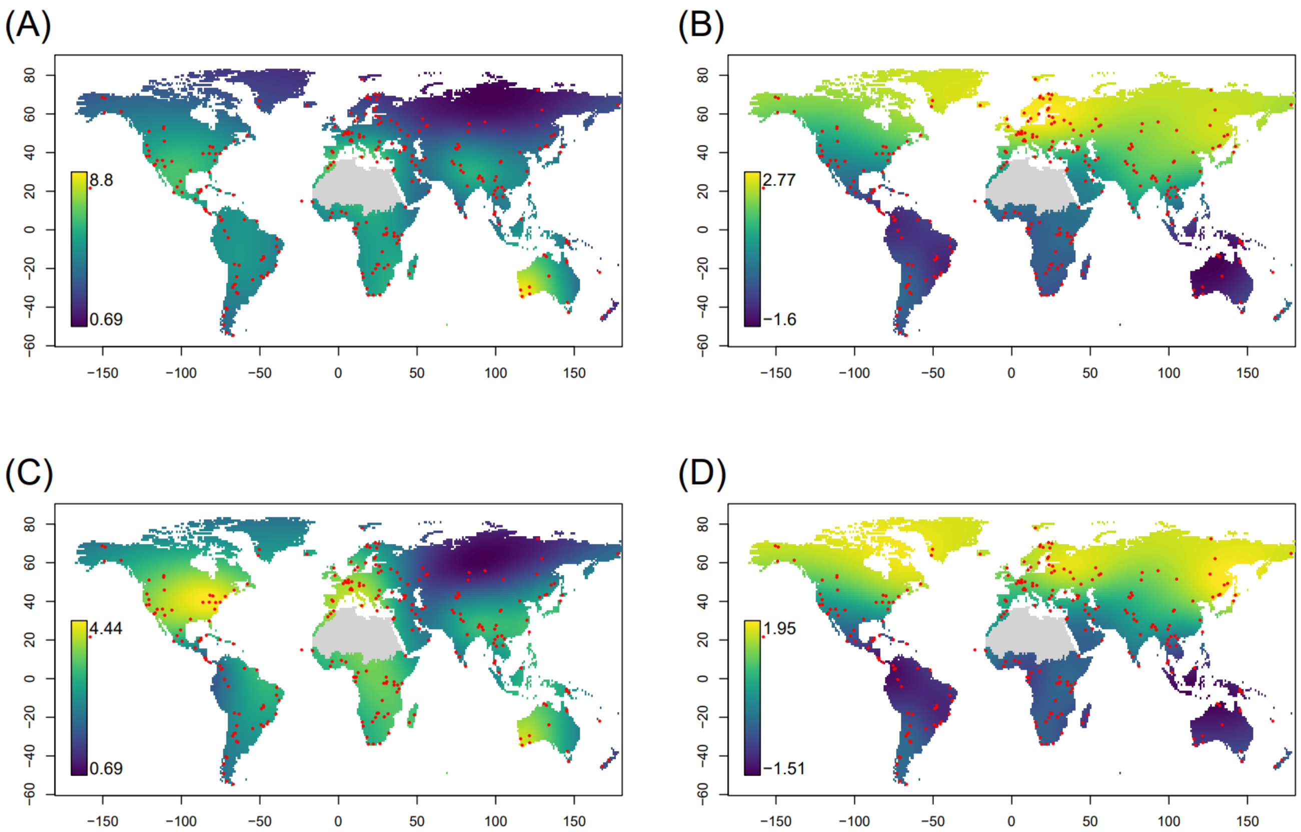

Supplementary Figure S2. Representation of taxa recorded in GBIF grid cells among taxa present among trnL sequence data in NCBI. Values are the logarithm of the ratio between the number of occurrences (based on the presence-absence) of taxa (A,B) or records (C,D) of families (A,C) and species (B,D) in a GBIF cell that are represented in NCBI and the number not represented. Global predictions are the result of a generalised additive model. Red points indicate sampling locations. The Sahara region was excluded from interpolations because of insufficient sampling.

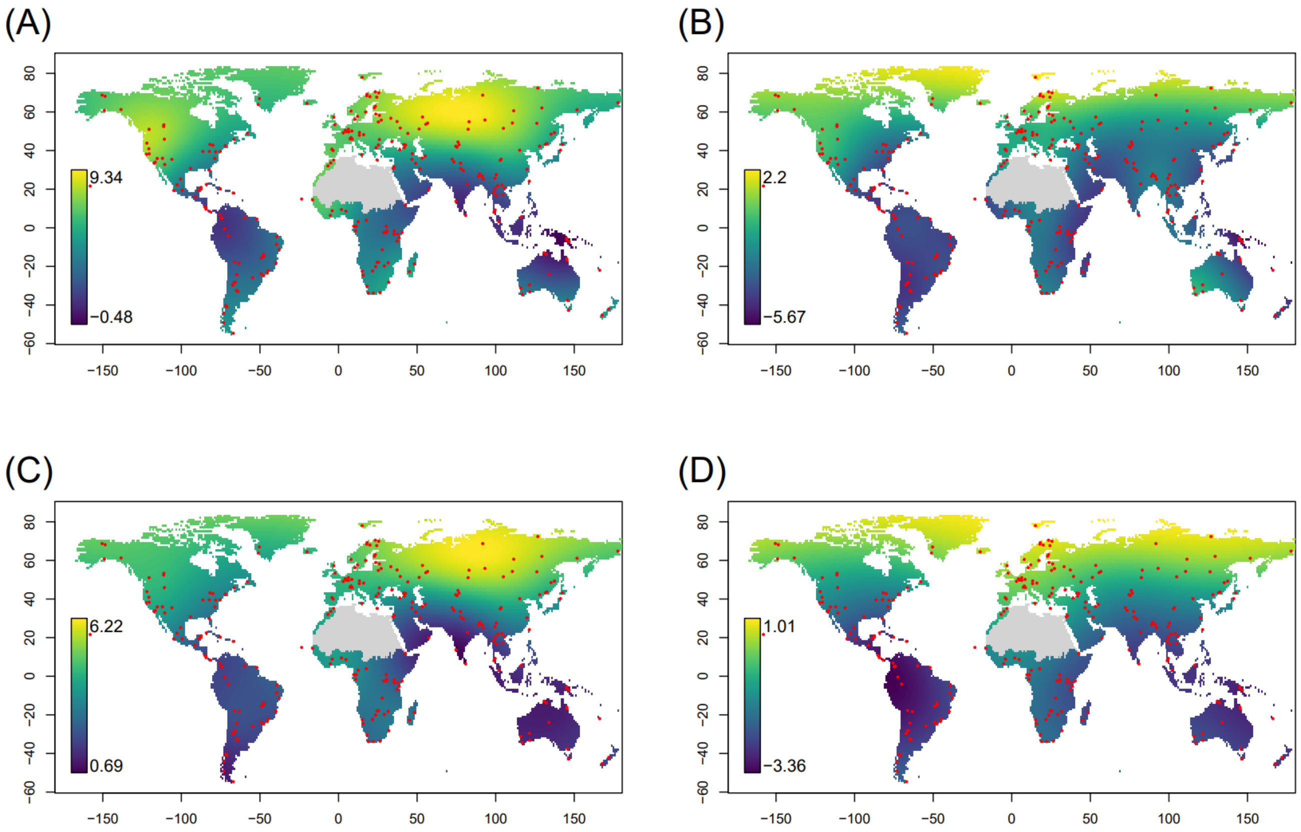

Supplementary Figure S3. Representation of taxa recorded in eDNA samples among corresponding GBIF data. Values are the logarithm of the ratio between the number of reads (A,B) or OTUs (C,D) representing families (A,C) or species (B,D) also present in GBIF and the number not present. Global predictions are the result of a generalised additive model. Red points indicate sampling locations. The Sahara region was excluded from interpolations because of insufficient sampling
